# Fingerprint Analysis of Volatile Flavor Compounds in *Crassostrea gigas* of Different Ploidy and Gender under High-Temperature Incubation

**DOI:** 10.3390/molecules28196857

**Published:** 2023-09-28

**Authors:** Youmei Sun, Jingjing Fu, Enshuo Zhang, Luyao Dong, Xuebo Cui, Yanan Sun, Zhizhong Wang, Yanwei Feng, Bin Li, Xiaohui Xu, Qihao Luo, Weijun Wang, Jianmin Yang

**Affiliations:** 1 School of Agriculture, Ludong University, Yantai 264025, China; sunyoumei4554@163.com (Y.S.); fjj000117@163.com (J.F.); zenshuo1998@163.com (E.Z.); c646175773@163.com (X.C.); fywzxm1228@163.com (Y.F.); xxh83121@163.com (X.X.); 2 College of Fisheries and Life Science, Shanghai Ocean University, Shanghai 201306, China; dly6568@163.com; 3 Yantai Kongtong Island Industrial Co., Ltd., Yantai 264000, China; 13305458716@163.com (B.L.); 17854266371@163.com (Q.L.); 4 Yantai Haiyu Marine Technology Co., Ltd., Yantai 264000, China

**Keywords:** *Crassostrea gigas*, GC-IMS, gender, ploidy, volatile flavor

## Abstract

In this study, diploid, triploid, and tetraploid *Crassostrea gigas* samples were subjected to gas chromatography and ion mobility (GC-IMS) to identify and analyze volatile compounds and flavor fingerprints under conditions of high-temperature incubation. The GC-IMS technology identified a total of 54 volatile components in *C. gigas*. The contents of 1-octen-3-ol, butyl pentanoate, p-methyl anisole, and 2-methyl-2-hepten-6-one in male oysters were significantly higher than in females, while the contents of phenylacetaldehyde, benzaldehyde, 2-ethyl-3-methylpyrazine, 2-ethylfuran, and 2,4-hexadienal in female oysters were significantly higher than in males. The contents of non-3-en-2-one-M and 1-pentanol in diploids were significantly higher than in triploids and tetraploids, while the content of 2,4-hexadienal in tetraploids was significantly higher than in diploids and tetraploids. The contents of ethyl acetate, ethyl-2-butenoate, and butanal in tetraploids were significantly higher than those in diploids and triploids. The results of a principal components analysis showed that different samples were relatively independently clustered, allowing the ability to distinguish different oyster samples. The chemical fingerprints of volatile compounds of *C. gigas* with different ploidy and gender under high-temperature incubation were established, and the volatile substance contours of *C. gigas* were visualized. The results provide a reference for distinguishing the ploidy and gender of *C. gigas* under conditions of high-temperature incubation.

## 1. Introduction

*Crassostrea gigas*, also known as the Pacific oyster, is the main cultured oyster species in northern China, where it is referred to as “sea milk” [1]. The qualities of rapid growth, strong adaptability, delicious meat quality, and nutrient richness have made oysters one of the most widely farmed shellfish in the world [2]. According to the fishery statistical yearbook of China, the total output of oysters reached 5.82 × 10^6^ t in 2021, accounting for 26.31% of the total output of marine aquaculture products and 38.13% of the total output of shellfish marine aquaculture products in China. There are diploid, triploid, and tetraploid oysters, and the most common oysters in nature are diploids, the type preferred by consumers. However, diploid oysters have a breeding period during early summer each year, and the generation and release of gametes result in poor meat quality and taste. Triploid oysters are a new variety formed by the hybridization of diploids and tetraploids. Due to chromosome instability in meiosis resulting in high sterility, the oysters display hypogonadal dysplasia and thus consume less energy for reproduction [3]. This mechanism makes triploid oysters allocate more energy to growth, resulting in larger individuals and hence better meat quality. The triploids have thus become a sterile form with commercial value [4], and they can fulfill the market shortage of diploid oysters in summer. Tetraploid oysters are obtained by inhibiting the polar body release of triploid oysters, a method employed in polyploid breeding. Tetraploid oysters are especially useful in the seed market, as they can be used by male parents to cross with female diploids to produce triploid oysters [5]. Neither triploids nor tetraploids exist in nature, but tetraploid oysters are fertile compared with triploids [6].

Oyster meat is rich in protein, glycogen, fats, and other substances that can be absorbed by the human body [7]. In addition, oysters are popular seafood because of their high quality and comparatively low price. Fresh oysters have a unique fragrance and are one of the few fresh animal foods eaten by humans [8]; therefore, the composition of volatile compounds figures prominently in their overall flavor. Studies have found that the nutrients in oysters can change during different periods [9]; the main nutritional components of oysters are distributed differently in different body parts [10], aquaculture areas, growth cycles and seasons, and varieties. These factors can greatly affect the biochemical composition and quality of oysters [11,12]. For consumers, the nutritional quality, functional factors, and flavor characteristics of oysters are important references for their processing and utilization. Therefore, studying the nutritional composition and chemical makeup of oysters is significant for the development of the oyster industry.

The flavor of food is economically important, as it can trigger consumers’ preferences. The flavor of food is recognized through specific sensory characteristics and can be used to evaluate the nutritional value of the food [13]. Gas chromatography and ion mobility (GC-IMS) is a new technology that can detect and identify volatile organic compounds (VOCs) in different substances. The principle of GC-IMS technology is to use gas chromatography to separate various compounds in the sample, and then the compounds are detected through ion migration spectroscopy technology. In gas chromatography, the mixture is injected into the gas chromatography column, and various compounds in the sample are separated by interaction with the column filler. Compounds move at different rates within the column based on their physical properties, thereby achieving separation. Finally, the compounds are separated from the column, and sent to an ion migration spectrometer for detection. This technology does not need pretreatment of the sample, and its operation is simple as well as low-cost. The method has been applied in many fields, including environmental analysis [14], medicine [15], and food [16] analysis. The volatile flavor compounds in aquatic foods have been analyzed. Twenty-six kinds of volatile components were identified in *Pseudosciaena crocea* oil, and with the extension of the storage period, the components and the number of oxidative substances increased, leading to the deterioration of fish oil [17]. When silver carp were inoculated with the dominant bacteria, 1-propanol, butanone, methyl isobutyl ketone, and dimethyl sulfide gradually increased with the extension of storage time, indicating that these compounds could possibly be used as markers for monitoring preservation or deterioration [18]. The volatile components of crab meat were characterized by solid-phase microextraction and gas chromatography-tandem spectroscopy, revealing that the relative contents of 3-methylbutyralde-hyde, heptanaldehyde, and benzaldehyde contributed to improving the flavor [19]. However, there are few reports concerning the identification of volatile flavor compounds in oysters. Previous studies have shown that the content of nonvolatile compounds in diploid oysters was higher than in triploid oysters, and the content of volatile compounds in diploid oysters was lower than that in triploid oysters [20]. There is also reports on fresh oysters of different ploidy and gender to analyze the changes in volatile components under room temperature conditions [21]. At present, little is known about the impact of high-temperature incubation on the flavor of oysters. Therefore, this study demonstrated the fingerprint of volatile flavor compounds at the high temperature condition, and compared the flavor of oysters under room temperature with high temperature incubation. The results of the study could help to show the difference of ploidies and genders of cooked oyster with different odors, which could increase the cooking value of oysters, thus expanding the consumer market for oysters and their application in food processing, storage and quality enhancement.

## 2. Results and Discussion

### 2.1. Analysis of GC-IMS Topographic Plots in Oysters of Different Ploidy and Gender

**Figure 1:**
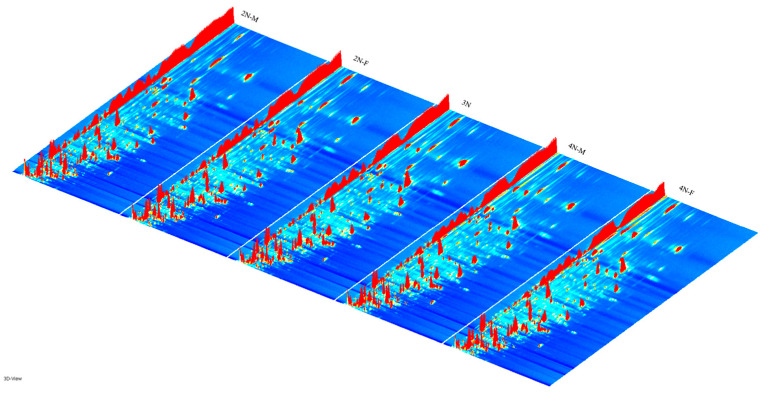
Three-dimensional topographic maps of different oyster samples. (2N-F: diploid females, 2N-M: diploid males, 3N: triploids, 4N-F: tetraploid females, 4N-M: tetraploid males).

**Figure 2:**
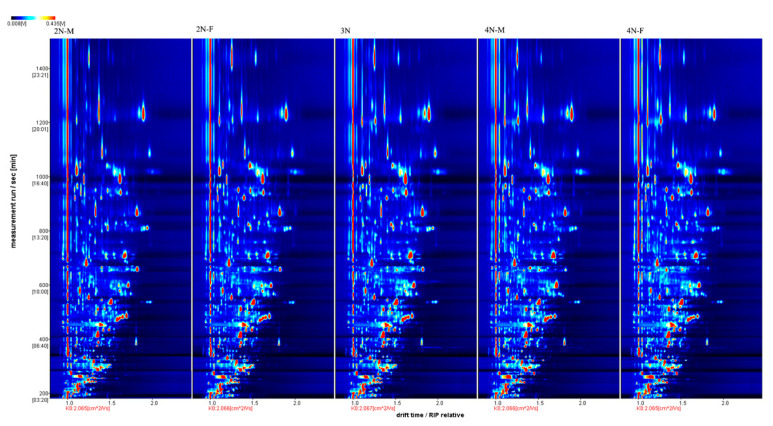
Two-dimensional spectra of different oyster samples.

**Figure 3:**
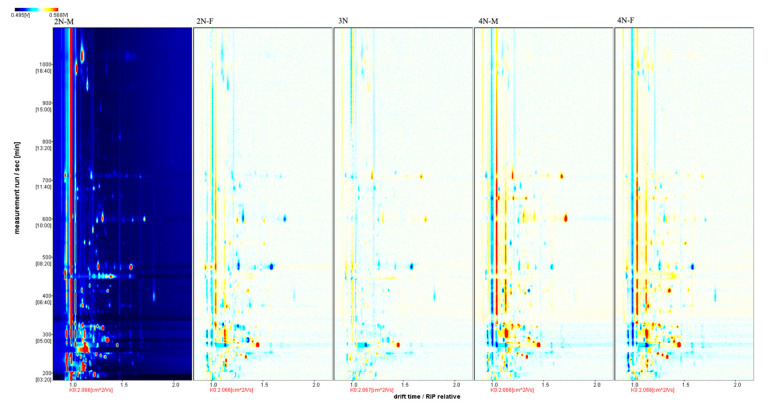
Comparison and differences in the spectra of volatile components of different oyster samples (using the 2N-M sample as a reference).

The three-dimensional spectra of volatile matter information of oysters with different ploidy are shown in Figure 1. In the figure, the *x*-axis represents the ion migration time; the *y*-axis represents the retention time of gas chromatographic; and the *z*-axis represents the peak intensity [22]. The three-dimensional spectrogram could intuitively represent the differences in VOCs in different samples. From the spectrogram, there were certain similarities in volatile compounds between samples, but the graph does not accurately identify samples that have greater contents of volatile compounds. Two-dimensional spectra of different oyster samples are shown in Figure 1. The results showed that the peak signal distribution of volatile compounds among oysters of different ploidy was similar, but the peak signal intensity in each sample was different, indicating variation in the content of volatile compounds. The two-dimensional spectrum of volatile components in the samples is shown in Figure 2. In the figure, the top view shows the three-dimensional spectrum, and the types and concentrations of volatile components in different samples can be noted. The red vertical line at abscissa 1.0 represents the RIP peak (reaction ion peak, normalized), and the bright spots on both sides represent volatile substances. Each point represents a substance. White indicates a low content of a substance, and red indicates a high concentration of the substance. As shown in Figure 2, the migration time of each substance was generally in the range of 1.0–2.0 s, and the chromatographic retention time was in the range of 200–1000 s. A comparison chart of differences is shown in Figure 3. The chart visually shows the differences in substance contents. Compared with other groups using diploid males (2N-M) as the reference group, if the composition was consistent, this is indicated by a white background, red indicates a higher substance content than the reference group, and blue indicates a lower substance content than the reference group. As shown in Figure 3, the volatile substances of *C. gigas* varied with ploidy.

### 2.2. Qualitative Analysis of Flavor Components of Oysters with Different Ploidy and Gender under High Temperature

**Figure 4:**
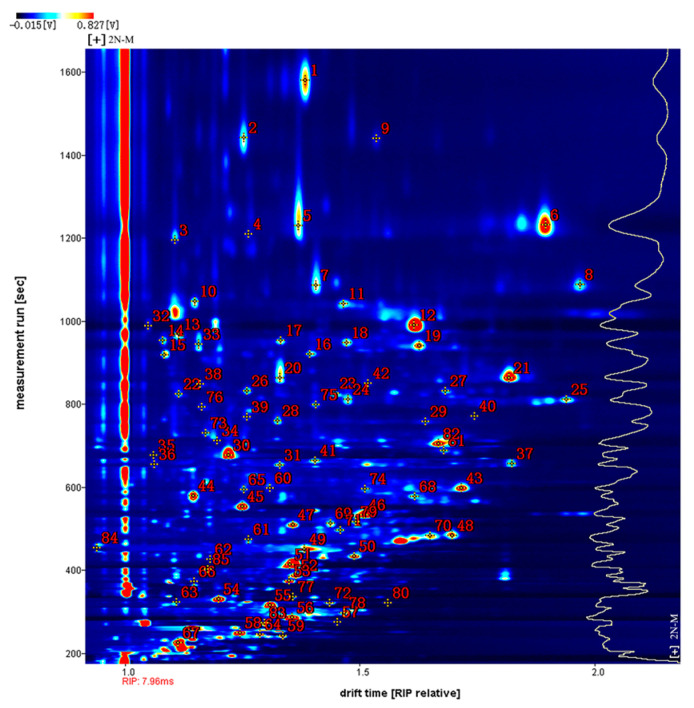
Qualitative analysis of VOCs in different oyster samples. (Note: Spectrum signals are represented by numbers).

Figure 4 shows the qualitative analysis results of the database search. The number next to the signal peak in the figure represents a specific flavor compound. Using the NIST (National Insititute of Standards and Technology and IMS migration time database with 2N-M as an example, a total of 85 signal peaks and 54 volatile compounds were detected (Table 1). Due to the limited data in the database, some compounds were not identified [23]. Among the identified compounds, there were 21 aldehydes, 12 ketones, eight alcohols, five esters, three furans, two acids, two pyrazines, and one ether. Since high concentrations of monomer ions and neutral molecules may form dimers in the drift region, a single compound may produce multiple signals, i.e., a monomer (M) and dimer (D) of the same compound. These included phenylacetaldehyde, propanoic acid, (E)-non-3-en-2-one, (E)-2-nonenal, benzaldehyde, furfural, 3-(methylsulfanyl) propanal, (E)-2-octenal, (E,E)-2,4-hexadienal, 1-nonanal, 1-hexanol, 2-butanone, 3-hydroxy, 3-octanone, (Z)-4-heptenal, and 1-pentanol. The volatile components detected are listed in Table 1.

**Figure 5:**
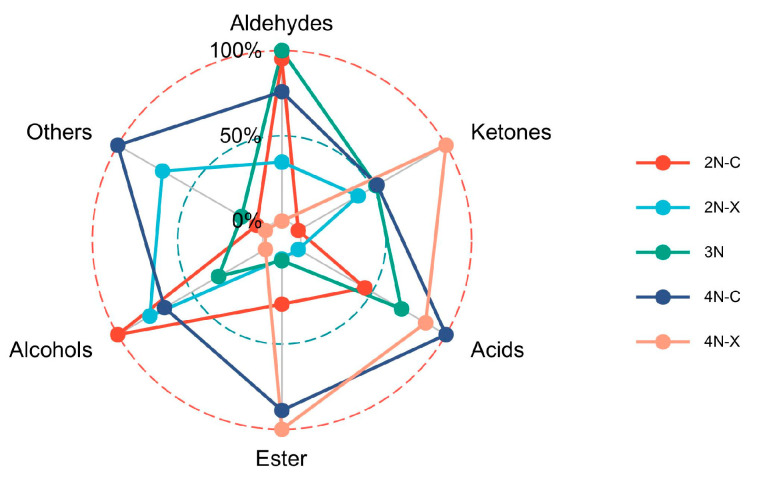
Comparison of VOCs in oysters from different samples.

Aldehydes, ketones, alcohols, and esters were the main components of VOCs in the oyster samples. As shown in Figure 5, the tetraploid oysters show an increase in volatile components compared to diploid and triploid oysters, which suggests that the flavors of oysters differ between ploidy. The content of esters was higher in diploid females than in diploid males, and the content of ketones was higher in diploid males than in diploid females. There were also visual differences between the gender of the tetraploid oysters, which suggests a difference in flavor between the gender.

### 2.3. Fingerprint Analysis of VOCs in Oysters under High Temperature

**Figure 6:**
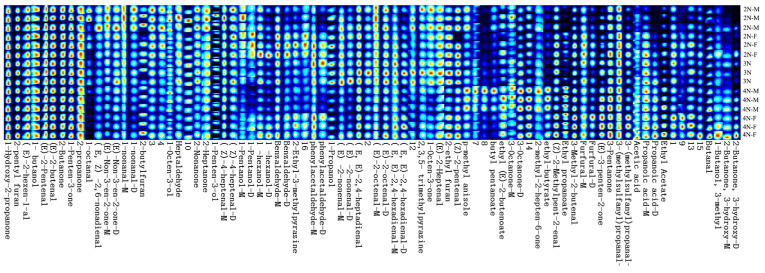
Fingerprints of VOCs in oysters with different ploidy and gender based on GC-IMS (Note: digit numbers under the figure indicate the unidentified compounds).

To more intuitively distinguish the patterns in VOCs in different oysters under high-temperature conditions, the Callery plot plugin for the laboratory analytical viewer (LAV) software was used to generate the fingerprints, as shown in Figure 6. Each horizontal row represents all of the volatile flavor substances in each sample, and each vertical column represents the content of the same VOCs in different samples. The colors of the dots represent the contents of the compounds. The redder the color, the higher the content.

In comparing the fingerprint spectra under room temperature conditions [21], the contents of ethyl (E)-2-butenoate, 1-propanol, 1-penten-3-ol, ethyl propanoate, and others decreased under high-temperature conditions, while the contents of 2-heptanone, 2-butanone, (E)-2-butenone, (E)-3-penten-2-one, (Z)-2-pentenal, (E)-2-pentenal, (Z)-2-methylpent-2-enal, heptaldehyde, and other substances increased, consistent with previous research results [24]. After high-temperature incubation, the fishy taste of oysters is weakened, and a strong shellfish aroma is produced. High-temperature conditions will intensify the oxidation of fatty acids, the Maillard reaction, and protein degradation in oysters, effects that may be the reason for the formation of oyster flavor after heating at 100 °C [25].

Aldehydes were the most volatile compounds isolated and identified from *C. gigas.* The aldehydes could be divided into saturated aldehydes and unsaturated aldehydes, and these may be produced from the cracking of peroxides formed after the oxidation of unsaturated fatty acids [26]. The higher the temperature, the greater the reaction intensity and the more aldehydes produced. This may be the reason for more aldehydes being produced under high-temperature incubation conditions [27]. For example, 1-nonanal and heptanaldehyde were derived from the oxidation of unsaturated fatty acids; these have the odor of grass and are the main products of ω-6 fatty acid peroxide degradation [28]. (E, E)-2,4-heptadienaldehyde contributes to the fragrance, fat aroma, vegetable aroma, and greasy odor. These compounds contribute to the overall flavor of oysters, being primarily responsible for the vegetable aroma and fat aroma in the meat.

Ketones are volatile components detected in oysters, and they are formed from the oxidative degradation of unsaturated fatty acids and the oxidation of alcohols [29]. Ketones in meat products usually are produced by the automatic oxidation of lipids [30], and they can also be formed through lipid decomposition during fermentation. The ketones detected in oysters were 3-octanone, 2-heptanone, 2-nonone, 2-propanone, and 2-butanone. The characteristic flavor of ketones is usually described as a creamy or cheese-like flavor [31].

The degradation of linoleic acid through oxidation can produce the alcohols. Alcohols have high thresholds and make a small contribution to flavor, but unsaturated alcohols have a low threshold and make a significant contribution to the flavor. For example, 1-octene-3-ol is commonly found in the volatile flavor compounds of fish, with a flavor reminiscent of mushrooms and vegetables. Due to its low threshold, it is one of the reasons for the peculiar odor [32]. In addition, 1-octene-3-ol has an earthy flavor, a clam flavor, and other flavors, and was present in both diploid and triploid oysters with similar contents [33].

Esters are formed by the esterification of inorganic or organic acids with alcohols to condense water. Most esters are major components of spices and have the aroma of fruits [34], a factor that could help the oysters produce a pleasant taste. Esters are common in fermented foods, and they are an important source of flavor substances in liquors. Esters that were detected in the oysters included ethyl butyrate, ethyl valerate, ethyl propionate, and ethyl acetate, compounds that were detected in tetraploid *C. gigas*, and there was a significant difference (*p* < 0.05) between these and the diploid and triploid oysters.

Furans have often been the focus of aroma research. Furan compounds are the early products of the Maillard reaction (MR) and are the precursors of nitrogen-containing heterocyclic compounds [35] that give food a caramel and baking flavor, and they made a positive contribution to the oyster flavor. There were three furans detected in this study, 2-pentylfuran, 2-ethyl furan, and 2-butyl furan, and there was no significant difference regarding the ploidy of oysters (*p* < 0.05). Acids yield a spicy and sour taste [36]. Pyrazines are derived from the MR between amino acids and carbohydrates. This can accelerate the production of pyrazines under high-temperature incubation [37]. Only one volatile ether was detected in this study. Furan and pyrazine are heterocyclic compounds; although their contents were low, their odor thresholds were also low, and thus, they made a significant contribution to the overall sensory flavor of oysters.

In summary, the overall flavor of the oyster was grassy, esterase fragrant and fruity. In conjunction with Figure 5, aldehydes were high in diploid and triploid oysters and relatively low in tetraploid oysters, and acids were high in tetraploid oysters and low in diploid and triploid oysters, so that diploid and triploid oysters had a better overall flavor than tetraploid oysters under high-temperature incubation conditions, and was better suited to be used in food cooking and processing.

### 2.4. PCA of Oysters with Different Gender and Ploidy

**Figure 7:**
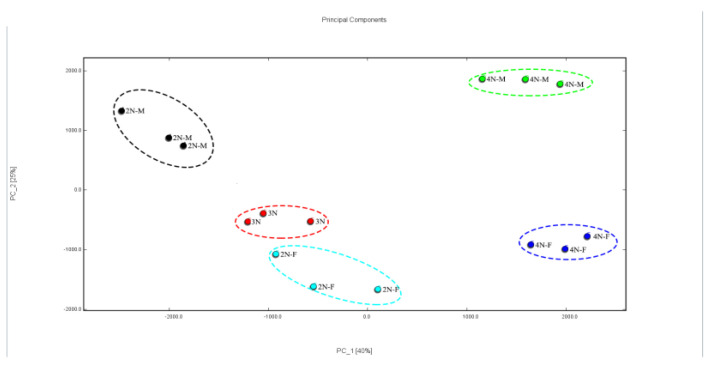
Principal component analysis of VOCs in different oyster samples.

Principal component analysis (PCA) is an important dimensionality reduction classification method [38]. This method has been widely used in the study of chemical fingerprints. The PCA analysis is shown in Figure 7. The total contributions of PC1 and PC2 were 40% and 25%, respectively. After dimensionality reduction, the cumulative contribution of the first two principal components was more than 65%. After feature compression, more information was retained that could characterize the feature differences of the original data. In terms of the degree of aggregation and dispersion of samples, the tetraploid samples were well separated and had apparent differences in characteristics. The difference in characteristics between diploids and triploids was not significant, especially between 3N and 2N-F.

The fingerprints of VOCs in oysters with different ploidy were analyzed. The VOCs in diploid and triploid oysters were similar, especially between female and triploid oysters. However, there was a significant difference between diploid and tetraploid oysters, a result that was consistent with the distinction made by the PCA analysis. Therefore, GC-IMS combined with PCA could better distinguish the VOCs in oysters regarding ploidy and gender.

### 2.5. Analysis of Volatile Compounds in Oysters between Males and Females

There were differences in volatile flavor compounds between males and females (Table 1). In diploids, the contents of females were significantly higher than those of males (*p* < 0.05). These compounds included phenylacetaldehyde, propanoic acid-M, benzaldehyde, 3-(methylsulfanyl) propanal, furfural, 2,4-hexadienal, 2-ethyl-3-methylpyrazine, 3-methyl-2-butenal, 2-methylpent-2-enal, 3-pentanone, ethyl propanoate, 1-pentanol, and 2-ethyl furan. The contents in males were significantly higher than those of females for non-3-en-2-one-M, 2,4-heptadienal, p-methyl anisole, 2-octenal-M, 1-nonanal, 1-octen-3-ol, butyl pentanoate, 3-octanone-M, 2-heptanone, 2-methyl-2-hepten-6-one, and 2-nonanone. In tetraploids, the contents in females that were significantly higher than in males included phenylacetaldehyde, benzaldehyde, furfural, 2,4-hexadienal, 1-hexanol, 2-ethyl-3-methylpyrazine, 3-octanone, 2-hexen-1-al, 2-methylpent-2-enal, 2-pentenal, 1-pentanol, and 2-ethyl furan. The contents in males were significantly higher than those in females for non-3-en-2-one, p-methyl anisole, 1-octen-3-ol, butyl pentanoate, 4-heptenal, 3-octanone, 2-methyl-2-hepten-6-one, and ethyl-butyrate. Under high-temperature conditions, there were significant differences in volatile flavor compounds between female and male oysters, possibly due to variations in the biochemical composition of male and female oyster gonads during development. Female individuals had higher glycogen, fat, Cu, and Zn content and lower protein content, a pattern related to the special meiosis mode of female oysters. Male individuals had significantly higher taurine and protein content than female individuals and higher nutritional quality, a result that may also be related to the regulation of gene expression in different oysters. These factors may have led to significant differences in the volatile flavor compounds in oysters of different genders [39,40].

### 2.6. Analysis of Volatile Compounds in Oysters of Different Ploidy

There were differences in volatile flavor compounds in oysters of different ploidy (Table 2). For triploids, compared with diploid and tetraploid male oysters, the contents of 2,6-nonadienal, 1-nonanal, 1-hydroxy-2-propanone, 1-octen-3-ol, 1-pentanol, 2-heptanone, and non-3-en-2-one-M in diploids were significantly higher than in triploids and tetraploids. The contents of phenylacetaldehyde, 2,4-heptadienal, 2,4-hexadienal, 2-hexen-1-al, 2-methylpent-2-enal, and ethyl-butyrate in triploids were significantly higher than in diploids and tetraploids. The contents of 3-octanone-D, 3-pentanone, ethyl propanoate, ethyl acetate, ethyl-2-butenoate, butanal, and 2-methyl-2-hepten-6-one in tetraploid were significantly higher than in diploids and triploids. In comparison of triploids with diploid and tetraploid female oysters, we found that the contents of non-3-en-2-one-M, benzaldehyde-M, and 1-pentanol in diploids were significantly higher than in triploids and tetraploids. The contents of 2,4-heptadienal, 2,4-hexadienal-M, 3-octanone-M, and 2-methyl-2-hepten-6-one in triploids were significantly higher than in diploids and tetraploids. The contents of propanoic acid-D, 2-nonenal-D, benzaldehyde-D, 3-hydroxy-D, 2-butanone, 3-hydroxy-M, 3-penten-2-one, ethyl propanoate, ethyl acetate, ethyl-2-butenoate, butanal, and ethyl-butyrate in tetraploids were significantly higher than in diploids and triploids.

Triploid *C. gigas* were sterile, and thus the main energy materials in the body could not be consumed and released in large quantities during the breeding period. Nevertheless, although tetraploid *C. gigas* are polyploid oysters, they can generate gametes during the breeding season, and they are usually more active than diploids [41]. These factors contributed to the differences in VOCs in oysters with different ploidy.

## 3. Materials and Methods

### 3.1. Materials

The diploid oyster samples used in the experiment were Luyi No.1 (Certificate No.: GS-006-2020) and were cultured at the Kongtong Island research base in Yantai, Shandong Province. Both triploids and tetraploids were bred from Luyi No. 1 and were collected in August 2022. All of the oyster samples were cultured in the same area and were at the same age of 14 months. The samples were divided into five groups: diploid females (2N-F), diploid males (2N-M), triploids (3N), tetraploid females (4N-F), and tetraploid males (4N-M). First, we determined the gender of the oysters. Oyster samples were dissected, and gametes were taken from gonad tissue and placed on microscope slides with a drop of water and observed under a microscope. A foggy appearance indicated a male and a granular appearance indicated a female. Next, we determined the ploidy of the sample. One gill filament was dissected and immediately cleaned with PBS buffer, then placed in a 1.5 mL centrifuge tube with 0.5 mL of PBS buffer. The gill filaments were cut with scissors, and 10 μL of 0.05 mg/mL DAPI(4′,6-Diamidino-2-phenylindole dihydrochloride) staining solution was added. The sample was mixed well and allowed to stand in the dark for 20 min. After staining, the supernatant was extracted and placed in a 2 mL centrifuge tube, and the ploidy of *C. gigas* was detected by flow cytometry (Beckman Coulter Life Sciences, Shanghai, China).

About 250 g of soft tissue (excluding the adductor muscle) from five oysters was taken in each group. A homogenizer was used to homogenize the products, and these were put into Ziplock bags and placed in a −80 °C refrigerator for freezing storage. The volatile components of each sample were identified using a Flavor Spec flavor analyzer (G.A.S, IMSPEX Diagnostics Ltd., Rhondda Cynon Taff, UK). All samples were analyzed in the same laboratory.

### 3.2. GC-IMS Injection Methods and Conditions

The volatile compounds in the *C. gigas* samples were analyzed using a GC–IMS (Flavorspec, G.A.S. Instrument, Dortmund, Germany). Before the experiments, the samples were thawed overnight (12 h) in the refrigerator at 4 °C. Without any pretreatment, 2 g samples were taken and placed in 20 mL headspace injection bottles for about 15 min of incubation. The injection conditions were set as follows: the injection volume was 700 μL; the incubation temperature was 100 °C; and the injection probe temperature was 105 °C. A gas chromatographic column was used (model: MXT-WAX, column length: 15 m, inner diameter: 0.53 mm, thickness: 1 μm). The column temperature was 60 °C. The IMS temperature was 45°C; the drift gas flow rate was 150 mL/min; the time was 30 min; the carrier gas flow rate was 2 (0–10 min), 10 (10–20 min), and 100 mL/min (20–30 min); and the carrier gas and drift gas were high purity nitrogen (purity 99.999%). Each group included three parallel samples.

### 3.3. Identification of Flavouring Substances

The volatile substances in oysters were qualitatively analyzed by comparing the retention times and migration rates of flavor substances with the GC-IMS database.

### 3.4. Statistical Analysis

The analysis required the use of configuration software and plug-ins in the GC-IMS instrument. The GC-IMS spectrum was analyzed by LAV software. The NIST (National Institute of Standards and Technology) database and IMS (Information Management System) database built-in VOCal software (Version 0.4.03, GAS Deutschland, Dortmund, Germany) were used for qualitative analysis of compounds. The reporter plugin was used to directly compare the differences in spectra between samples. A Callery plot plugin was employed to compare fingerprints to more intuitively identify the differences of volatile substances between different sample groups. The dynamic PCA plugin performed a principal component analysis to detect the signal strengths of volatile compounds to highlight the differences between samples. We used the hiplot online analysis tool (https://hiplot.cn) to construct radar charts that more visually show the variation of different substances from sample to sample. Independent sample *t*-tests and single-factor ANOVA in SPSS software (SPSS Inc., Chicago, IL, USA) were used to analyze the differences between samples (*p* < 0.05 was considered a significant difference).

## 4. Conclusions

In this study, GC-IMS was used to analyze the volatile compounds in oysters with different ploidy and gender. A total of 54 volatile components were detected, including monomers and dimers of several components. There were 21 aldehydes, 12 ketones, eight alcohols, five esters, three furans, two acids, two pyrazines, one ether, and some detected but undetermined components. These volatile components were the main contributors to the flavor of oysters, and the volatile components of five groups of oysters showed significant differences. Through fingerprinting, we are also able to visualize that 2-pentyl furan, hydroxy-2-propanone, (E)-2-hexen-1-al, 1-penten-3-one, 2-butanone were in the similar level in five groups of samples. However, octanal, 1-nonanal, 2-butylfuran, and heptanal were the highest in diploid male samples, 1-pentanol, 1-hexanol, benzaldehyde, and phenylacetaldehyde with the same condition in diploid female samples. (E)-2-nonenal, (E)-2-octenal, and (E)-2-heptenal were the highest in triploids. Butyl pentanoate, 3-octanone, 2-methyl-2- hepten-6-one, and ethyl-butyrate were the highest in tetraploid male samples, and furfural, (E)-3-penten-2-one, acetic acid, propionic acid, and 3-hydroxy-2-butanone were the highest in tetraploid females. In addition, the results of the PCA showed that different samples were in relatively independent spaces and could thus be distinguished. This study established the fingerprints of volatile compounds in oysters with different ploidy and gender under high-temperature incubation. The types and contents of flavor compounds were significantly increased compared with the profiles under ambient conditions, which suggested that high-temperature incubation could cause oysters to exude more flavors and enhance the taste. For consumers, the taste of oyster is the most important, so diploid and triploid oysters fragrant taste is more suitable for consumers; another important reason is its market price, tetraploid oyster is only used for breeding or seeds, which does not exist in nature at present, and their price is quite high, so diploid and triploid oysters with a lower price are more popular among consumers. This study can provide a theoretical basis for studying the characteristics of oysters with different ploidy and gender, especially concerning their flavor differences, and also provides ideas for subsequent research, thus expanding the application and practical value of GC-IMS analysis in the field of food processing.

## Figures and Tables

**Table 1:** GC-IMS-based identification of differences between male and female oysters.

No.	Compounds	Retention Indexwas ^①^	Retention Times/s ^②^	Drift Times/ms ^③^	The Peak Volume			
					2N-M	2N-F	4N-M	4N-F
1	2,6-Nonadienal	1737.5	1580.877	1.3853	3614.95 ± 625.89 ^a^	2638.27 ± 215.09 ^a^	2398.55 ± 207.31 ^a^	2417.17 ± 562.71 ^a^
2	Phenylacetaldehyde-M	1691.5	1441.904	1.25563	2962.37 ± 540.43 ^b^	4720.72 ± 380.44 ^a^	3398.64 ± 374.22 ^b^	4814.85 ± 436.66 ^a^
3	Propanoic acid-M	1597.9	1195.785	1.10796	702.04 ± 208.09 ^b^	1769.18 ± 231.90 ^a^	1988.80 ± 61.07 ^a^	1951.52 ± 24.93 ^a^
4	Propanoic acid-D	1603.6	1209.576	1.26464	259.47 ± 33.18 ^a^	1096.23 ± 513.26 ^a^	2697.14 ± 353.55 ^a^	3326.19 ± 468.83 ^a^
5	Non-3-en-2-one-M	1611.9	1229.733	1.37089	4502.48 ± 195.95 ^a^	3313.85 ± 254.12 ^b^	2358.76 ± 216.17 ^a^	1877.52 ± 56.64 ^b^
6	Non-3-en-2-one-D	1612.7	1231.854	1.89675	5894.10 ± 999.21 ^a^	5180.27 ± 544.60 ^a^	4681.11 ± 424.71 ^a^	3966.77 ± 212.65 ^a^
7	2-Nonenal-M	1549.9	1086.517	1.40871	2203.23 ± 445.26 ^a^	2478.60 ± 128.62 ^a^	1643.50 ± 180.85 ^a^	1761.12 ± 366.59 ^a^
8	2-Nonenal-D	1550.3	1087.265	1.96933	1155.34 ± 442.15 ^a^	1497.46 ± 176.40 ^a^	797.45 ± 110.38 ^a^	1081.54 ± 463.80 ^a^
9	Phenylacetaldehyde-D	1691.2	1441.125	1.53643	352.22 ± 134.27 ^b^	931.06 ± 163.63 ^a^	426.08 ± 77.59 ^b^	916.71 ± 149.26 ^a^
10	Benzaldehyde-M	1531.9	1048.106	1.1505	1066.71 ± 76.65 ^b^	1440.78 ± 33.15 ^a^	1019.22 + 34.12 ^b^	1187.27 ± 91.02 ^a^
11	Benzaldehyde-D	1529.3	1042.566	1.46691	929.69 ± 181.52 ^b^	2123.12 ± 159.67 ^a^	1095.86 ± 34.85 ^b^	1731.84 ± 148.59 ^a^
12	2,4-Heptadienal	1503.8	990.859	1.6172	9300.60 ± 949.89 ^a^	9600.97 ± 288.00 ^a^	9119.32 ± 194.01 ^a^	9774.12 ± 538.55 ^a^
13	P-methyl anisole	1491.9	967.468	1.1157	1073.96 ± 115.51 ^a^	702.07 ± 34.32 ^b^	1194.09 ± 106.49 ^a^	862.66 ± 68.33 ^b^
14	Furfural-M	1484.8	953.925	1.08248	524.73 ± 24.34 ^b^	749.10 ± 17.31 ^a^	585.03 ± 27.45 ^b^	713.18 ± 21.01 ^a^
15	3-(Methylsulfanyl)propanal-M	1466.1	918.838	1.08564	1087.48 ± 99.84 ^b^	1416.57 ± 51.71 ^a^	1238.97 ± 29.93 ^a^	1317.54 ± 47.63 ^a^
16	3-(Methylsulfanyl)propanal-D	1467.4	921.301	1.39572	927.84 ± 189.84 ^b^	2754.40 ± 249.13 ^a^	1714.15 ± 134.28 ^b^	3148.18 ± 541.38 ^a^
17	Furfural-D	1484.2	952.694	1.33244	634.25 ± 142.30 ^b^	1465.46 ± 297.48 ^a^	1020.68 ± 208.12 ^a^	1889.78 ± 570.98 ^a^
18	2-Octenal-M	1434.9	863.438	1.33086	2236.74 ± 98.58 ^a^	1919.40 ± 30.01 ^b^	1586.58 ± 140.09 ^a^	1495.10 ± 131.17 ^a^
19	2-Octenal-D	1434.9	863.438	1.81812	4870.75 ± 601.37 ^a^	4536.22 ± 45.17 ^a^	3664.12 ± 437.59 ^a^	4084.97 ± 724.36 ^a^
20	2,4-Hexadienal-M	1411.6	824.042	1.1157	364.24 ± 14.61 ^b^	453.00 ± 25.17 ^a^	372.98 ± 13.43 ^b^	430.13士25.58 ^a^
21	2,4-Hexadienal-D	1413.1	826.505	1.44793	669.31 ± 207.58 ^a^	983.80 ± 122.06 ^a^	778.13 ± 103.80 ^b^	1339.22 ± 142.67 ^a^
22	1-Nonanal-M	1403.7	811.116	1.4764	889.80 ± 74.43 ^a^	746.71 ± 28.38 ^b^	577.41 ± 47.94 ^a^	528.30 ± 79.21 ^a^
23	1-Nonanal-D	1404.0	811.731	1.94152	1067.88 ± 62.11 ^a^	642.53 ± 19.66 ^b^	511.37 ± 62.42 ^a^	436.47 ± 147.38 ^a^
24	1 -Hexanol-M	1370.7	759.409	1.32611	643.96 ± 84.80 ^a^	769.35 ± 208.89 ^a^	401.64 ± 25.94 ^b^	669.21 ± 118.30 ^a^
25	1 -Hexanol-D	1369.5	757.562	1.64093	263.18 ± 68.64 ^a^	499.62 ± 258.50 ^a^	226.00 ± 24.56 ^b^	635.29 ± 157.76 ^a^
26	1-Hydroxy-2-propanone	1313.6	677.539	1.2217	7858.05 ± 124.31 ^a^	7808.35 ± 156.96 ^a^	7622.40 ± 106.25 ^a^	7710.54 ± 227.09 ^a^
27	2-Butanone, 3-hydroxy-D	1294.8	652.276	1.3317	303.62 ± 317.69 ^a^	744.15 ± 702.64 ^a^	985.74 ± 428.39 ^a^	1683.33 ± 569.71 ^a^
28	Acetic acid	1502.6	988.507	1.05136	376.67 ± 79.83 ^a^	586.05 ± 103.68 ^a^	693.37 ± 81.45 ^a^	748.35 ± 95.55 ^a^
29	1-Octen-3-ol	1479.7	944.205	1.1587	1268.69 ± 126.73 ^a^	882.65 ± 101.45 ^b^	1010.72 ± 24.29 ^a^	748.78 ± 98.92 ^b^
30	2-Butanone, 3-hydroxy-M	1296.6	654.891	1.06375	16.09 ± 10.35 ^a^	25.55 ± 11.43 ^a^	46.99 ± 14.53 ^a^	55.26 + 5.36 ^a^
31	1-Octanal	1298.7	657.566	1.82562	2021.51 ± 880.19 ^a^	1225.73 ± 615.38 ^a^	433.54 ± 216.37 ^a^	242.30 ± 68.23 ^a^
32	2-Ethyl-3-methylpyrazine	1426.5	849.028	1.16209	182.46 ± 28.86 ^b^	345.41 ± 34.67 ^a^	205.64 ± 45.11 ^b^	330.52 ± 60.83 ^a^
33	Butyl pentanoate	1304.3	665.014	1.40639	697.33 ± 80.72 ^a^	400.51 ± 56.36 ^b^	948.68 ± 286.92 ^a^	430.91 ± 116.68 ^b^
34	3-Octanone-D	1265.7	598.497	1.71809	2962.69 ± 372.31 ^a^	2318.53 ± 311.79 ^a^	6043.67 ± 276.33 ^b^	4207.17 ± 401.51 ^a^
35	4-Heptenal-M	1254.7	579.319	1.14747	3077.19 ± 279.21 ^a^	2777.98 ± 56.12 ^a^	2592.92 ± 90.69 ^a^	2320.46 ± 88.89 ^b^
36	2-Pentyl furan	1239.0	552.949	1.25109	4519.06 ± 422.30 ^a^	4321.01 ± 395.99 ^a^	4193.73 ± 106.37 ^a^	4596.74 ± 373.05 ^a^
37	2-Hexen-1-al	1227.3	534.114	1.51085	6289.12 ± 255.17 ^a^	6409.53 ± 197.19 ^a^	6276.38 ± 37.11 ^b^	6662.21 ± 163.24 ^a^
38	3-Methyl-2-butenal	1211.6	509.799	1.35897	2267.89 ± 501.95 ^b^	4204.71 ± 224.04 ^a^	3866.57 ± 433.44 ^a^	4730.05 ± 528.16 ^a^
39	Heptaldehyde	1194.1	484.114	1.69822	1776.15 ± 254.45 ^a^	1542.15 ± 95.40 ^a^	1243.67 ± 70.05 ^a^	1111.35 ± 206.21 ^a^
40	1-Butanol	1169.8	450.824	1.38425	2307.16 ± 140.27 ^a^	2514.14 ± 28.74 ^a^	2475.51 ± 36.51 ^a^	2496.20 ± 41.12 ^a^
41	2-Methylpent-2-enal	1157.3	434.743	1.48969	1645.24 ± 212.60 ^b^	2206.08 ± 211.15 ^a^	1548.84 ± 112.00 ^b^	2322.87 ± 142.34 ^a^
42	2-Pentenal-M	1139.8	413.076	1.35223	9105.77 ± 573.14 ^a^	9380.81 ± 90.28 ^a^	8989.74 ± 132.24 ^b^	9472.37 ± 186.12 ^a^
43	2-Pentenal-D	1118.0	387.568	1.36614	1907.72 ± 214.96 ^a^	1600.01 ± 1210.54 ^a^	1613.89 ± 99.93 ^b^	2144.46 ± 212.64 ^a^
44	3-Penten-2-one	1105.9	374.154	1.35167	831.67 ± 483.01 ^a^	971.24 ± 196.12 ^a^	2144.30 ± 297.76 ^a^	2590.21 ± 476.93 ^a^
45	2-Butenal	1053.9	331.065	1.20029	3801.89 ± 390.19 ^a^	4067.09 ± 128.18 ^a^	3800.42 ± 44.57 ^a^	3871.73 ± 292.41 ^a^
46	1-Penten-3-one	1031.9	315.212	1.31048	4784.76 ± 255.40 ^a^	4746.80 ± 119.90 ^a^	4109.79 ± 81.19 ^a^	4314.03 ± 239.13 ^a^
47	3-Pentanone	985.5	285.945	1.35723	4949.16 ± 557.67 ^b^	5107.29 ± 1075.45 ^a^	6090.46 ± 152.37 ^a^	6186.13 ± 611.14 ^a^
48	Ethyl propanoate	961.9	274.969	1.45295	221.58 ± 93.33 ^b^	861.17 ± 56.26 ^a^	1584.98 ± 54.30 ^a^	1631.41 ± 139.55 ^a^
49	2-Butanone	901.0	248.547	1.24704	3733.68 ± 357.01 ^a^	4144.13 ± 105.49 ^a^	3718.13 ± 115.57 ^a^	3807.81 ± 289.43 ^a^
50	Ethyl Acetate	884.0	241.637	1.33831	595.45 ± 30.41 ^a^	1023.52 ± 268.18 ^a^	1605.95 ± 266.81 ^a^	1865.56 ± 397.52 ^a^
51	3-Octanone-M	1265.2	597.553	1.30973	343.27 ± 52.48 ^a^	212.85 ± 8.45 ^b^	378.17 ± 11.18 ^a^	268.86 ± 14.02 ^b^
52	Ethyl-2-butenoate	1150.4	426.075	1.18305	83.01 ± 20.17 ^a^	84.70 ± 37.84 ^a^	143.85 ± 23.22 ^a^	134.98 ± 9.06 ^a^
53	1-Propanol	1043.2	323.287	1.11199	282.56 ± 60.58 ^a^	272.82 ± 65.20 ^a^	182.39 ± 26.25 ^a^	178.56 ± 9.58 ^a^
54	Butanal	896.2	246.591	1.28862	535.51 ± 41.09 ^a^	609.33 ± 93.65 ^a^	817.93 ± 87.41 ^a^	885.59 ± 118.60 ^a^
55	1-Pentanol-M	1263.7	594.974	1.25483	284.90 ± 38.66 ^a^	263.16 ± 20.01 ^a^	116.72 ± 9.41 ^b^	161.17 ± 6.73 ^a^
56	2-Propanone	843.9	226.112	1.11453	4972.37 ± 338.98 ^a^	5443.53 ± 37.48 ^a^	5267.57 ± 12.94 ^a^	5386.67 ± 121.03 ^a^
57	4-Heptenal-D	1254.0	578.092	1.61885	684.27 ± 34.63 ^a^	690.08 ± 18.96 ^a^	608.60 ± 19.80 ^a^	545.10 ± 76.57 ^a^
58	2-Heptanone	1193.4	483.056	1.65086	1303.53 ± 126.14 ^a^	1059.84 ± 49.67 ^b^	1129.27 ± 36.97 ^a^	977.68 ± 93.08 ^b^
59	2-Methyl-2-hepten-6-one	1351.1	730.198	1.17369	180.69 ± 21.18 ^a^	131.14 ± 8.37 ^b^	224.38 ± 20.80 ^a^	151.93 ± 10.63 ^b^
60	1-Pentanol-D	1264.4	596.137	1.51151	256.29 + 10.65 ^b^	388.89 ± 22.99 ^a^	108.87 ± 12.94 ^b^	226.49 ± 45.69 ^a^
61	2-Nonanone	1396.2	799.107	1.40828	356.63 ± 13.84 ^a^	317.17 ± 13.22 ^b^	228.48 ± 43.89 ^a^	228.86 ± 35.64 ^a^
62	2,3,5-Trimethylpyrazine	1393.1	794.156	1.16446	240.04 ± 30.66 ^a^	199.72 ± 11.88 ^a^	195.91 ± 32.88 ^a^	198.56 ± 24.83 ^a^
63	1-Butanol, 3-methyl	1216.4	517.124	1.49232	147.21 ± 19.68 ^a^	290.34 ± 144.07 ^a^	264.46 ± 98.02 ^a^	467.26 ± 130.57 ^a^
64	Ethyl-butyrate	1040.9	321.634	1.56149	80.48 ± 3.18 ^a^	92.61 ± 7.40 ^a^	347.08 ± 9.62 ^a^	240.48 ± 40.62 ^b^
65	1-Octen-3-one	1321.3	688.033	1.67936	260.60 ± 30.85 ^a^	219.09 ± 25.34 ^a^	128.62 ± 42.40 ^a^	156.76 ± 14.54 ^a^
66	2-Heptenal	1333.5	704.915	1.6689	1857.34 ± 128.86 ^a^	1888.34 ± 78.53 ^a^	1593.03 ± 72.01 ^a^	1823.52 ± 163.56 ^a^
67	2-Ethyl furan	957.8	273.08	1.29858	546.04 ± 100.35 ^b^	720.80 ± 58.29 ^a^	477.96 ± 6.32 ^b^	719.17 ± 46.26 ^a^
68	1-Penten-3-ol	1171.9	453.687	0.94218	454.13 ± 154.65 ^a^	342.80 ± 58.29 ^a^	397.91 ± 20.56 ^a^	350.62 ± 22.42 ^a^
69	2-Butylfuran	1130.9	402.512	1.17811	210.79 ± 67.27 ^a^	171.31 ± 113.98 ^a^	163.78 ± 19.68 ^a^	212.76 ± 48.90 ^a^

Note: ^a,b^ Means within the same row indicate statistical differences significantly (*p* < 0.05). ^①^ Represents the retention index calculated using n-ketones C4–C9 as external references. ^②^ Represents the retention time in the capillary GC column. ^③^ Represents the time in the drift tube.

**Table 2:** GC-IMS-based identification of differences between diploid, triploid, and tetraploid oysters.

No.	Compounds	The Peak Volume					
		2N-M	3N	4N-M	2N-F	3N	4N-F
1	2,6-Nonadienal	3614.95 ± 625.89 ^a^	2298.87 ± 230.63 ^b^	2398.55 ± 207.31 ^b^	2638.27 ± 215.09 ^a^	2298.87 ± 230.63 ^a^	2417.17 ± 562.71 ^a^
2	Phenylacetaldehyde-M	2962.37 ± 540.43 ^b^	4634.13 ± 434.03 ^a^	3398.64 ± 374.22 ^b^	4720.72 ± 380.44 ^a^	4634.13 ± 434.03 ^a^	4814.85 ± 436.66 ^a^
3	Propanoic acid-M	702.04 ± 208.09 ^b^	1951.87 ± 53.57 ^a^	1988.80 ± 61.07 ^a^	1769.18 ± 231.90 ^a^	1951.87 ± 53.57 ^a^	1951.52 ± 24.93 ^a^
4	Propanoic acid-D	259.47 ± 33.18 ^b^	2141.86 ± 33.18 ^a^	2697.14 ± 353.55 ^a^	1096.23 ± 513.26 ^b^	2141.86 ± 33.18 ^b^	3326.19 ± 468.83 ^a^
5	Non-3-en-2-one-M	4502.48 ± 195.95 ^a^	2837.78 ± 297.98 ^b^	2358.76 ± 216.17 ^b^	3313.85 ± 254.12 ^a^	2837.78 ± 297.98 ^b^	1877.52 ± 56.64 ^c^
6	Non-3-en-2-one-D	5894.10 ± 999.21 ^a^	5683.71 ± 836.84 ^a^	4681.11 ± 424.71 ^a^	5180.27 ± 544.60 ^a^	5683.71 ± 836.84 ^a^	3966.77 ± 212.65 ^b^
7	2-Nonenal-M	2203.23 ± 445.26 ^a b^	2625.30 ± 242.17 ^a^	1643.50 ± 180.85 ^b^	2478.60 ± 128.62 ^a^	2625.30 ± 242.17 ^a^	1761.12 ± 366.59 ^b^
8	2-Nonenal-D	1155.34 ± 442.15 ^a b^	1760.11 ± 414.91 ^a^	797.45 ± 110.38 ^b^	797.45 ± 110.38 ^b^	797.45 ± 110.38 ^b^	1081.54 ± 463.80 ^a^
9	Phenylacetaldehyde-D	352.22 ± 134.27 ^b^	991.89 ± 229.70 ^a^	426.08 ± 77.59 ^b^	931.06 ± 163.63 ^a^	991.89 ± 229.70 ^a^	916.71 ± 149.26 ^a^
10	Benzaldehyde-M	1066.71 ± 76.65 ^a b^	1183.71 ± 79.64 ^a^	1019.22 + 34.12 ^b^	1440.78 ± 33.15 ^a^	1183.71 ± 79.64 ^b^	1187.27 ± 91.02 ^b^
11	Benzaldehyde-D	929.69 ± 181.52 ^b^	1194.05 ± 100.39 ^a^	1095.86 ± 34.85 ^a b^	1095.86 ± 34.85 ^b^	1194.05 ± 100.39 ^b^	1731.84 ± 148.59 ^a^
12	2,4-Heptadienal	1731.84 ± 148.59 ^b^	11318.15 ± 743.89 ^a^	9119.32 ± 194.01 ^b^	9119.32 ± 194.01 ^b^	11318.15 ± 743.89 ^a^	9774.12 ± 538.55 ^b^
13	P-methyl anisole	1073.96 ± 115.51 ^a^	848.60 ± 69.60 ^b^	1194.09 ± 106.49 ^a^	702.07 ± 34.32 ^b^	848.60 ± 69.60 ^a^	862.66 ± 68.33 ^a^
14	Furfural-M	524.73 ± 24.34 ^a^	664.54 ± 127.56 ^a^	585.03 ± 27.45 ^a^	749.10 ± 17.31 ^a^	664.54 ± 127.56 ^a^	713.18 ± 21.01 ^a^
15	3-(Methylsulfanyl)propanal-M	1087.48 ± 99.84 ^b^	1328.43 ± 85.16 ^a^	1238.97 ± 29.93 ^a b^	1416.57 ± 51.71 ^a^	1328.43 ± 85.16 ^a^	1317.54 ± 47.63 ^a^
16	3-(Methylsulfanyl)propanal-D	927.84 ± 189.84 ^b^	1969.60 ± 420.36 ^a^	1714.15 ± 134.28 ^a^	2754.40 ± 249.13 ^a b^	1969.60 ± 420.36 ^b^	3148.18 ± 541.38 ^a^
17	Furfural-D	634.25 ± 142.30 ^a^	1097.83 ± 564.94 ^a^	1020.68 ± 208.12 ^a^	1465.46 ± 297.48 ^a^	1097.83 ± 564.94 ^a^	1889.78 ± 570.98 ^a^
18	2-Octenal-M	2236.74 ± 98.58 ^a^	2040.14 ± 24.93 ^a^	1586.58 ± 140.09 ^b^	1919.40 ± 30.01 ^a^	2040.14 ± 24.93 ^a^	1495.10 ± 131.17 ^b^
19	2-Octenal-D	4870.75 ± 601.37 ^a^	5159.56 ± 329.39 ^a^	3664.12 ± 437.59 ^b^	4536.22 ± 45.17 ^a b^	5159.56 ± 329.39 ^a^	4084.97 ± 724.36 ^b^
20	2,4-Hexadienal-M	364.24 ± 14.61 ^b^	506.11 ± 5.44 ^a^	372.98 ± 13.43 ^b^	453.00 ± 25.17 ^b^	506.11 ± 5.44 ^a^	430.13士25.58 ^b^
21	2,4-Hexadienal-D	669.31 ± 207.58 ^b^	1252.48 ± 270.00 ^a^	778.13 ± 103.80 ^b^	983.80 ± 122.06 ^a^	1252.48 ± 270.00 ^a^	1339.22 ± 142.67 ^a^
22	1-Nonanal-M	889.80 ± 74.43 ^a^	777.07 ± 16.48 ^b^	577.41 ± 47.94 ^c^	746.71 ± 28.38 ^a^	777.07 ± 16.48 ^a^	528.30 ± 79.21 ^b^
23	1-Nonanal-D	1067.88 ± 62.11 ^a^	706.59 ± 64.48 ^b^	511.37 ± 62.42 ^c^	642.53 ± 19.66 ^a^	706.59 ± 64.48 ^a^	436.47 ± 147.38 ^b^
24	1 -Hexanol-M	643.96 ± 84.80 ^a^	619.10 ± 90.91 ^a^	401.64 ± 25.94 ^b^	769.35 ± 208.89 ^a^	619.10 ± 90.91 ^a^	669.21 ± 118.30 ^a^
25	1 -Hexanol-D	263.18 ± 68.64 ^a^	295.86 ± 43.48 ^a^	226.00 ± 24.56 ^a^	499.62 ± 258.50 ^a^	295.86 ± 43.48 ^a^	635.29 ± 157.76 ^a^
26	1-Hydroxy-2-propanone	7858.05 ± 124.31 ^a^	7596.07 ± 90.84 ^b^	7622.40 ± 106.25 ^b^	7808.35 ± 156.96 ^a^	7596.07 ± 90.84 ^a^	7710.54 ± 227.09 ^a^
27	2-Butanone, 3-hydroxy-D	303.62 ± 317.69 ^b^	508.76 ± 201.58 ^a b^	985.74 ± 428.39 ^a^	744.15 ± 702.64 ^b^	508.76 ± 201.58 ^b^	1683.33 ± 569.71 ^a^
28	Acetic acid	376.67 ± 79.83 ^b^	520.46 ± 139.03 ^a b^	693.37 ± 81.45 ^a^	586.05 ± 103.68 ^a^	520.46 ± 139.03 ^a^	748.35 ± 95.55 ^a^
29	1-Octen-3-ol	1268.69 ± 126.73 ^a^	953.30 ± 152.12 ^b^	1010.72 ± 24.29 ^b^	882.65 ± 101.45 ^a^	953.30 ± 152.12 ^a^	748.78 ± 98.92 ^a^
30	2-Butanone, 3-hydroxy-M	16.09 ± 10.35 ^b^	28.85 ± 12.95 ^a b^	46.99 ± 14.53 ^a^	25.55 ± 11.43 ^b^	28.85 ± 12.95 ^b^	55.26 + 5.36 ^a^
31	1-Octanal	2021.51 ± 880.19 ^a^	1341.53 ± 293.02 ^a b^	433.54 ± 216.37 ^b^	1225.73 ± 615.38 ^a^	1341.53 ± 293.02 ^a^	242.30 ± 68.23 ^b^
32	2-Ethyl-3-methylpyrazine	182.46 ± 28.86 ^b^	258.66 ± 19.11 ^a^	205.64 ± 45.11 ^a b^	345.41 ± 34.67 ^a^	258.66 ± 19.11 ^b^	330.52 ± 60.83 ^a b^
33	Butyl pentanoate	697.33 ± 80.72 ^a b^	455.52 ± 12.51 ^b^	948.68 ± 286.92 ^a^	400.51 ± 56.36 ^a^	455.52 ± 12.51 ^a^	430.91 ± 116.68 ^a^
34	3-Octanone-D	2962.69 ± 372.31 ^c^	4273.72 ± 160.13 ^b^	6043.67 ± 276.33 ^a^	2318.53 ± 311.79 ^b^	4273.72 ± 160.13 ^a^	4207.17 ± 401.51 ^a^
35	4-Heptenal-M	3077.19 ± 279.21 ^a^	2799.16 ± 71.53 ^a b^	2592.92 ± 90.69 ^b^	2777.98 ± 56.12 ^a^	2799.16 ± 71.53 ^a^	2320.46 ± 88.89 ^b^
36	2-Pentyl furan	4519.06 ± 422.30 ^a^	4280.96 ± 316.60 ^a^	4193.73 ± 106.37 ^a^	4321.01 ± 395.99 ^a^	4280.96 ± 316.60 ^a^	4596.74 ± 373.05 ^a^
37	2-Hexen-1-al	6289.12 ± 255.17 ^b^	6850.33 ± 144.46 ^a^	6276.38 ± 37.11 ^b^	6409.53 ± 197.19 ^b^	6850.33 ± 144.46 ^a^	6662.21 ± 163.24 ^a b^
38	3-Methyl-2-butenal	2267.89 ± 501.95 ^b^	2980.58 ± 390.69 ^a b^	3866.57 ± 433.44 ^a^	4204.71 ± 224.04 ^a^	2980.58 ± 390.69 ^b^	4730.05 ± 528.16 ^a^
39	Heptaldehyde	1776.15 ± 254.45 ^a^	1587.65 ± 115.73 ^a^	1243.67 ± 70.05 ^b^	1542.15 ± 95.40 ^a^	1587.65 ± 115.73 ^a^	1111.35 ± 206.21 ^b^
40	1-Butanol	2307.16 ± 140.27 ^a^	2456.76 ± 75.36 ^a^	2475.51 ± 36.51 ^a^	2514.14 ± 28.74 ^a^	2456.76 ± 75.36 ^a^	2496.20 ± 41.12 ^a^
41	2-Methylpent-2-enal	1645.24 ± 212.60 ^b^	2125.66 ± 165.99 ^a^	1548.84 ± 112.00 ^b^	2206.08 ± 211.15 ^a^	2125.66 ± 165.99 ^a^	2322.87 ± 142.34 ^a^
42	2-Pentenal-M	9105.77 ± 573.14 ^a^	9677.83 ± 207.16 ^a^	8989.74 ± 132.24 ^a^	9380.81 ± 90.28 ^a^	9677.83 ± 207.16 ^a^	9472.37 ± 186.12 ^a^
43	2-Pentenal-D	1907.72 ± 214.96 ^a b^	2129.03 ± 371.07 ^a^	1613.89 ± 99.93 ^b^	1600.01 ± 1210.54 ^a^	2129.03 ± 371.07 ^a^	2144.46 ± 212.64 ^a^
44	3-Penten-2-one	831.67 ± 483.01 ^b^	1396.78 ± 783.10 ^a b^	2144.30 ± 297.76 ^a^	971.24 ± 196.12 ^b^	1396.78 ± 783.10 ^b^	2590.21 ± 476.93 ^a^
45	2-Butenal	3801.89 ± 390.19 ^a^	4128.85 ± 142.12 ^a^	3800.42 ± 44.57 ^a^	4067.09 ± 128.18 ^a^	4128.85 ± 142.12 ^a^	3871.73 ± 292.41 ^a^
46	1-Penten-3-one	4784.76 ± 255.40 ^a^	4558.64 ± 285.28 ^a b^	4109.79 ± 81.19 ^b^	4746.80 ± 119.90 ^a^	4558.64 ± 285.28 ^a^	4314.03 ± 239.13 ^a^
47	3-Pentanone	4949.16 ± 557.67 ^b^	5080.89 ± 217.52 ^b^	6090.46 ± 152.37 ^a^	5107.29 ± 1075.45 ^a^	5080.89 ± 217.52 ^a^	6186.13 ± 611.14 ^a^
48	Ethyl propanoate	221.58 ± 93.33 ^c^	402.61 ± 96.42 ^b^	1584.98 ± 54.30 ^a^	861.17 ± 56.26 ^b^	402.61 ± 96.42 ^c^	1631.41 ± 139.55 ^a^
49	2-Butanone	3733.68 ± 357.01 ^a^	3962.26 ± 104.31 ^a^	3718.13 ± 115.57 ^a^	4144.13 ± 105.49 ^a^	3962.26 ± 104.31 ^a^	3807.81 ± 289.43 ^a^
50	Ethyl Acetate	595.45 ± 30.41 ^b^	717.56 ± 34.30 ^b^	1605.95 ± 266.81 ^a^	1023.52 ± 268.18 ^b^	717.56 ± 34.30 ^b^	1865.56 ± 397.52 ^a^
51	3-Octanone-M	343.27 ± 52.48 ^a b^	300.69 ± 21.73 ^b^	378.17 ± 11.18 ^a^	212.85 ± 8.45 ^c^	300.69 ± 21.73 ^a^	268.86 ± 14.02 ^b^
52	ethyl-2-butenoate	83.01 ± 20.17 ^b^	79.01 ± 17.24 ^b^	143.85 ± 23.22 ^a^	84.70 ± 37.84 ^b^	79.01 ± 17.24 ^b^	134.98 ± 9.06 ^a^
53	1-Propanol	282.56 ± 60.58 ^a^	321.14 ± 22.58 ^a^	182.39 ± 26.25 ^b^	272.82 ± 65.20 ^a^	321.14 ± 22.58 ^a^	178.56 ± 9.58 ^b^
54	Butanal	535.51 ± 41.09 ^b^	548.74 ± 20.16 ^b^	817.93 ± 87.41 ^a^	609.33 ± 93.65 ^b^	548.74 ± 20.16 ^b^	885.59 ± 118.60 ^a^
55	1-Pentanol-M	284.90 ± 38.66 ^a^	194.14 ± 23.91 ^b^	116.72 ± 9.41 ^c^	263.16 ± 20.01 ^a^	194.14 ± 23.91 ^b^	161.17 ± 6.73 ^b^
56	2-Propanone	4972.37 ± 338.98 ^a^	5252.56 ± 99.67 ^a^	5267.57 ± 12.94 ^a^	5443.53 ± 37.48 ^a^	5252.56 ± 99.67 ^b^	5386.67 ± 121.03 ^a b^
57	4-Heptenal-D	684.27 ± 34.63 ^a^	643.55 ± 32.90 ^a b^	608.60 ± 19.80 ^b^	690.08 ± 18.96 ^a^	643.55 ± 32.90 ^a^	545.10 ± 76.57 ^b^
58	2-Heptanone	1303.53 ± 126.14 ^a^	1139.09 ± 43.70 ^b^	1129.27 ± 36.97 ^b^	1059.84 ± 49.67 ^a b^	1139.09 ± 43.70 ^a^	977.68 ± 93.08 ^b^
59	2-Methyl-2-hepten-6-one	180.69 ± 21.18 ^b^	184.64 ± 3.79 ^b^	224.38 ± 20.80 ^a^	131.14 ± 8.37 ^c^	184.64 ± 3.79 ^a^	151.93 ± 10.63 ^b^
60	1-Pentanol-D	256.29 + 10.65 ^a^	168.55 ± 23.99 ^b^	108.87 ± 12.94 ^c^	388.89 ± 22.99 ^a^	168.55 ± 23.99 ^b^	226.49 ± 45.69 ^b^
61	2-Nonanone	356.63 ± 13.84 ^a^	365.25 ± 18.84 ^a^	228.48 ± 43.89 ^b^	317.17 ± 13.22 ^a^	365.25 ± 18.84 ^a^	228.86 ± 35.64 ^b^
62	2,3,5-Trimethylpyrazine	240.04 ± 30.66 ^a^	253.86 ± 26.87 ^a^	195.91 ± 32.88 ^a^	199.72 ± 11.88 ^b^	253.86 ± 26.87 ^a^	198.56 ± 24.83 ^b^
63	1-Butanol, 3-methyl	147.21 ± 19.68 ^b^	190.88 ± 6.04 ^a b^	264.46 ± 98.02 ^a^	290.34 ± 144.07 ^a b^	190.88 ± 6.04 ^b^	467.26 ± 130.57 ^a^
64	Ethyl-butyrate	80.48 ± 3.18 ^b^	53.45 ± 1.58 ^a^	347.08 ± 9.62 ^c^	92.61 ± 7.40 ^b^	53.45 ± 1.58 ^b^	240.48 ± 40.62 ^a^
65	1-Octen-3-one	260.60 ± 30.85 ^a^	255.57 ± 12.47 ^a^	128.62 ± 42.40 ^b^	219.09 ± 25.34 ^a^	255.57 ± 12.47 ^a^	156.76 ± 14.54 ^b^
66	2-Heptenal	1857.34 ± 128.86 ^a^	2012.07 ± 75.74 ^a^	1593.03 ± 72.01 ^b^	1888.34 ± 78.53 ^a^	2012.07 ± 75.74 ^a^	1823.52 ± 163.56 ^a^
67	2-Ethyl furan	546.04 ± 100.35 ^b^	704.45 ± 71.84 ^a^	477.96 ± 6.32 ^b^	720.80 ± 58.29 ^a^	704.45 ± 71.84 ^a^	719.17 ± 46.26 ^a^
68	1-Penten-3-ol	454.13 ± 154.65 ^a^	400.02 ± 49.66 ^a^	397.91 ± 20.56 ^a^	342.80 ± 58.29 ^a^	400.02 ± 49.66 ^a^	350.62 ± 22.42 ^a^
69	2-Butylfuran	210.79 ± 67.27 ^a^	163.97 ± 69.67 ^a^	163.78 ± 19.68 ^a^	171.31 ± 113.98 ^a^	163.97 ± 69.67 ^a^	212.76 ± 48.90 ^a^

Note: ^a,b,c^ Means with different letters within the same row differ significantly (*p* < 0.05).

## Data Availability

Not applicable.

## Sample Availability

Samples of the compounds are not available from the authors.
